# Hospital sanitary facilities on wards with high antibiotic exposure play an important role in maintaining a reservoir of resistant pathogens, even over many years

**DOI:** 10.1186/s13756-023-01236-w

**Published:** 2023-04-15

**Authors:** Claudio Neidhöfer, Esther Sib, Marcel Neuenhoff, Oliver Schwengers, Tobias Dummin, Christian Buechler, Niklas Klein, Julian Balks, Katharina Axtmann, Katjana Schwab, Tobias A. W. Holderried, Georg Feldmann, Peter Brossart, Steffen Engelhart, Nico T. Mutters, Gabriele Bierbaum, Marijo Parčina

**Affiliations:** 1 Institute of Medical Microbiology, Immunology and Parasitology, University Hospital Bonn, Venusberg Campus 1, 53127 Bonn, Germany; 2 Institute for Hygiene and Public Health, University Hospital Bonn, Bonn, Germany; 3 Bioinformatics and Systems Biology, Justus Liebig University Giessen, Giessen, Germany; 4 Department of Microbiology and Hospital Hygiene, Bundeswehr Central Hospital Koblenz, Koblenz, Germany; 5 Department of Oncology, Hematology, and Rheumatology, University Hospital Bonn, Bonn, Germany

**Keywords:** Multidrug-resistant bacteria, Carbapenemase-encoding bacteria, Antibiotic-resistance, CRKP, CRPsA, *K. pneumoniae* ST147, *P. aeruginosa* ST823, Bacterial colonization, Hospital hygiene, Hospital wastewater

## Abstract

**Background:**

Hospitals with their high antimicrobial selection pressure represent the presumably most important reservoir of multidrug-resistant human pathogens. Antibiotics administered in the course of treatment are excreted and discharged into the wastewater system. Not only in patients, but also in the sewers, antimicrobial substances exert selection pressure on existing bacteria and promote the emergence and dissemination of multidrug-resistant clones. In previous studies, two main clusters were identified in all sections of the hospital wastewater network that was investigated, one *K. pneumoniae* ST147 cluster encoding NDM- and OXA-48 carbapenemases and one VIM-encoding *P. aeruginosa* ST823 cluster. In the current study, we investigated if NDM- and OXA-48-encoding *K. pneumoniae* and VIM-encoding *P. aeruginosa* isolates recovered between 2014 and 2021 from oncological patients belonged to those same clusters.

**Methods:**

The 32 isolates were re-cultured, whole-genome sequenced, phenotypically tested for their antimicrobial susceptibility, and analyzed for clonality and resistance genes in silico.

**Results:**

Among these strains, 25 belonged to the two clusters that had been predominant in the wastewater, while two others belonged to a sequence-type less prominently detected in the drains of the patient rooms.

**Conclusion:**

Patients constantly exposed to antibiotics can, in interaction with their persistently antibiotic-exposed sanitary facilities, form a niche that might be supportive for the emergence, the development, the dissemination, and the maintenance of certain nosocomial pathogen populations in the hospital, due to antibiotic-induced selection pressure. Technical and infection control solutions might help preventing transmission of microorganisms from the wastewater system to the patient and vice versa, particularly concerning the shower and toilet drainage. However, a major driving force might also be antibiotic induced selection pressure and parallel antimicrobial stewardship efforts could be essential.

## Introduction

Antibiotic resistance is a major global concern, a complex public health issue and is accelerated by improper use of antibiotics as well as a growing population and increased networking and travelling. It depends on many interconnected factors and is far from limited to the clinical setting [1–3]. Resistance is usually acquired through the uptake of resistance genes by bacterial conjugation or other horizontal transmission pathways, spontaneous mutation of genes, upregulation of efflux pumps or intrinsic resistance genes, which subsequently allow the spread of resistant clones by vertical propagation [4]. After administration, antibiotics and their metabolites are released through environmental matrices, such as the sewage system, and exert selection pressure on the bacteria in this environment, favoring the occurrence of the mechanisms described above, multiplying resistance in the environment [1, 5–8]. Among antibiotic-resistant bacteria, carbapenemase-encoding Gram-negative bacteria are currently the most critical microorganisms [9, 10]. Carbapenems are antibiotics of last resort and administered in life-threatening infections caused by Gram-negative bacteria. Because of the large number of high-risk patients in hemato-oncology departments, these only intravenously administered substances are used there to an inordinately high extent, and as a result are released in excessively large quantities into the same sanitary facilities. Such concentrations were found to be in therapeutic concentrations [11, 12]. Because of this massive use of last-resort antibiotics, these highly resistant and critical bacteria are spread primarily through hospital wastewater, as opposed to municipal wastewater or agricultural process water [13]. Furthermore antibiotic resistant bacteria in wastewater are often associated with the ability to form biofilms in which they are able to survive even when confronted with high levels of antibiotics or disinfectants [14].

*Klebsiella pneumoniae* and *Pseudomonas aeruginosa* are two of the most important opportunistic and nosocomial pathogens worldwide and are known for the ability to produce biofilms to escape treatment with antibiotics [3, 15, 16]. In this study, we compared NDM and OXA-48 encoding *K. pneumoniae* and VIM encoding *P. aeruginosa* isolates recovered from clinical specimens of patients in oncology wards of a tertiary care center to the highly resistant strains that most prominently colonized patient bathrooms or were recovered from different sampling points of the same sewer system. Environmental *K. pneumoniae* ST147 and *P. aeruginosa* ST823 were analyzed when encoding the mentioned carbapenemases [17]. *K. pneumoniae* ST147 is a well-known high-risk clone that likely emerged in the 1990s and swiftly became a prominent global pathogen [18–20]. Different ST147 clusters are associated with different carbapenemases [18]. On the other hand, *P. aeruginosa* ST823 is mostly associated with *bla*_VIM_ and only few publications exist [21–23]. Reports of this strain in Europe are limited [17, 24].

## Methods

### Isolates

In the period under study Gram-negative bacterial pathogens recovered from clinical specimens were routinely identified via MALDI-TOF MS (VITEK MS, Biomerieux, Marcy-l’Etoile, France) and susceptibility-tested with the VITEK 2 system (Biomerieux, Marcy-l’Etoile, France). Carbapenem-resistant *K. pneumoniae* and *P. aeruginosa* isolates, or such with an unusual carbapenem-susceptibility profile (ertapenem/imipenem/meropenem) were routinely analyzed for the presence of common resistance genes, using the Allplex Entero-DR Assay (Seegene, Seoul, South Korea).

All VIM-encoding *P. aeruginosa* isolates and all NDM- and OXA-48-encoding *K. pneumoniae* isolates recovered from patients of oncology wards between September 2014 and November 2021 were traced in the laboratory information system, thawed and sub-cultured twice on Columbia 5% sheep blood agar (Becton Dickinson, Heidelberg, Germany) prior to testing and DNA extraction. Only first isolates were selected for each pathogen–patient combination. The environmental strains used for comparison had been isolated between Nov. 2016 and Sept. 2018 [17]

### Susceptibility testing

Antimicrobial susceptibility of all isolates after re-cultivation from cryo stocks was determined thrice by broth microdilution. Susceptibility tests were employed strictly according to the manufacturer’s instruction. From each isolate, a bacterial suspension in 0.9% saline solution was prepared. The suspension was adjusted to a McFarland value of between 0.48 and 0.52 using a DensiCHEK plus photometer (bioMerieux, Marcy-l’Etoile, France). For broth microdilution, Micronaut-S MDR MRGN-Screening MIC-Plates (Merlin, Bornheim, Germany) were utilized (tested antibiotics are listed in the legend of Table 2 in the “Results” section). Tests were performed with Mueller–Hinton broth (Merlin, Bornheim, Germany) and read with a BioTek ELx808 Absorbance Microplate Reader (now: Agilent Technologies Inc., Santa Clara, CA, USA). MICs were interpreted according to EUCAST 2022 v12 breakpoints (version 01.01.2022) for *Enterobacterales* and *Pseudomonas*, respectively.

### Whole genome sequencing

Highly purified DNA was extracted from all strains using the column-based DNeasy UltraClean Microbial Kit (Qiagen GmbH, Hilden, Germany). The isolation was performed according to the manufacturer’s instructions. Obtained DNA was qualitatively and quantitatively evaluated using the NanoDrop OneC from Thermo Fisher Scientific Inc. (Waltham, MA, USA). Dual-indexed Illumina sequencing libraries were constructed from each sample using the Illumina Nextera XT DNA Library Preparation Kit, pooled, and sequenced on the Illumina MiSeq platform with the Illumina MiSeq Reagent Kit v3, 600 cycles (all three: Illumina, San Diego, CA, USA). All steps were carried out following the manufacturer's instructions. Raw reads have been uploaded to the Sequence Read Archive (SRA); accession PRJNA845217.

### Assembly and genome analysis

Genome assembly and analysis were carried out independently in two different ways. On the one hand, paired-end reads were trimmed and filtered with BBDuk Trimmer with a Q value of 20 and de novo assembled using Geneious Prime (software version 2020.1 Biomatters, Auckland, New Zealand). Analysis of the de novo assembled contigs was then performed with online tools of the CGE-server, ResFinder-4.0 (https://cge.cbs.dtu.dk/services/ResFinder/) and PlasmidFinder-2.0 (https://cge.cbs.dtu.dk/services/PlasmidFinder/) [25–28] and the AMRFinderPlus v3.10.24 (https://www.ncbi.nlm.nih.gov/pathogens/antimicrobial-resistance/AMRFinder/) (with its according NCBI reference databases of 04-04-2022 [29]), which were used to identify the presence of antimicrobial resistance genes [30]. In addition to BLASTX, nucleotide sequences were translated into amino-acid sequences to identify corresponding proteins with ALLELEX and EXACTX. Genes with insertions for a stop codon were found with INTERNAL_STOP. POINTN considered strain-specific point mutations and refer to the majority of mentioned stress factors. PARTIALX took gene fragments with incomplete reference sequence into account. The minimum coverage value for PARTIALX was set to 60%. Only results with > 90% identity and > 90% coverage were accepted. On the other hand, genomes were assembled and analyzed with ASA^3^P v1.3.0 [31].

### Clustering

For epidemiological clustering Ridom SeqSphere + (version 6.0.2) (Ridom; Münster, Germany) (http://www.ridom.de/seqsphere) [32] was used. *K. pneumoniae* sensu lato was employed as cgMLST template for the *K. pneumoniae* strains and the cgMLST template for *P. aeruginosa* was used for the *P. aeruginosa* strains (www.ridom.de/seqsphere/u/Task_Template_Sphere.html). Minimum spanning trees were calculated after ignoring pairwise missing values and after exclusion of genes that were present only in the template strain.

### Phylogenetic analysis

Raw sequencing reads for both *K. pneumoniae* and *P. aeruginosa* isolates were processed using ASA^3^P v1.3.0 [30]. SNP-based maximum-likelyhood phylogenetic trees were calculated with FastTree within ASA^3^P calculating 100 bootstraps using isolates CNK1 and A15448 [17] as reference genomes for the sets, respectively.

All data relevant to the study are included in the article or uploaded as Appendix. The precise source of each environmental sample can be located in the corresponding publication [17]. Environmental samples were collected in approximately equal quantities from drains of sinks, toilets, and showers of hospital rooms in the hemato-oncological ward, from the wastewater of the hemato-oncological clinic, and wastewater sampling locations downstream of it.

The ethics committee of the University Hospital Bonn confirmed that no ethics approval was required for this study.

## Results

### Isolate and patient information

From September 2014 until November 2021, twenty-two VIM-encoding *P. aeruginosa* isolates and ten NDM- and OXA48-encoding *K. pneumoniae* isolates from patients of the oncology clinic were included into this study. Typing revealed that the majority of *P. aeruginosa* isolates (17) belonged to ST823; two belonged to ST235, another two to ST111, and one to ST233 (see Table 1). All but two *K. pneumoniae* isolates (which belonged to ST78) belonged to ST147. In the majority of patients (18/32), the isolates were first detected after more than four weeks of hospitalization. In half of the patients (16/32), the isolates were obtained from stool samples. All but one, *K. pneumoniae* ST147 isolates, were recovered from the same ward.

**Table 1:**
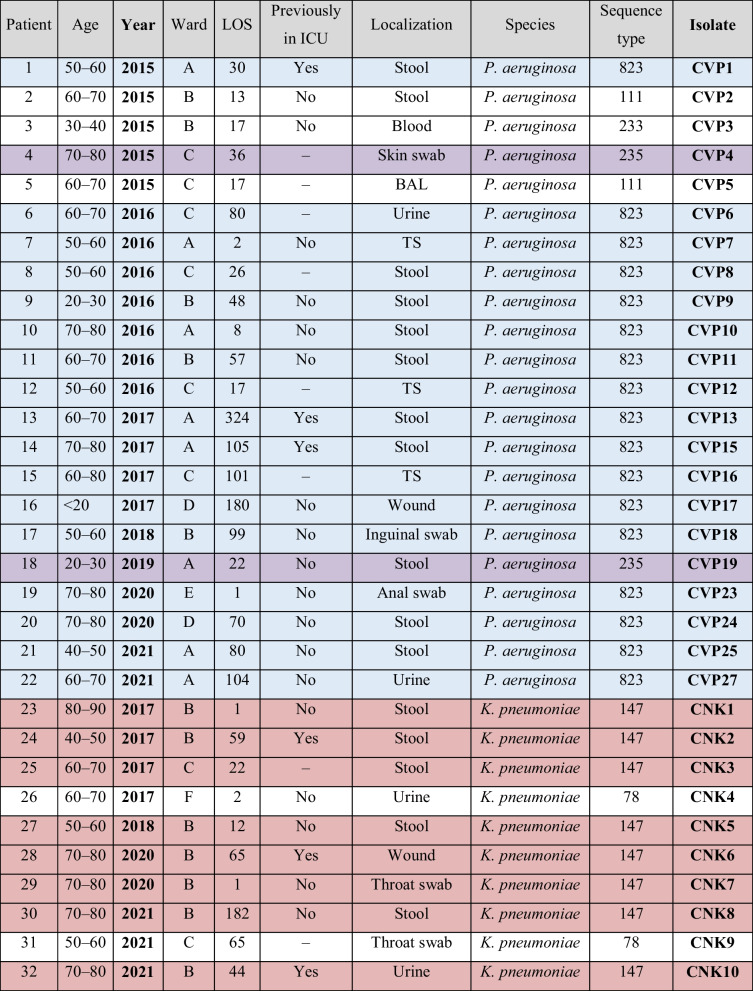
General patient information of genotyped VIM-encoding *P. aeruginosa* isolates (CVP) (above) and NDM- and OXA48-encoding *K. pneumoniae* isolates (CNK) (below)

*LOS*: Length of Hospital Stay in days, previous to the date on which the positive sample was collected. Rows are color-coded according to isolate sequence type for better visualization, *P. aeruginosa* ST823 cluster in light blue, *P. aeruginosa* ST235 cluster in purple and *K. pneumoniae* ST147 cluster in red

### Clustering

**Fig. 1:**
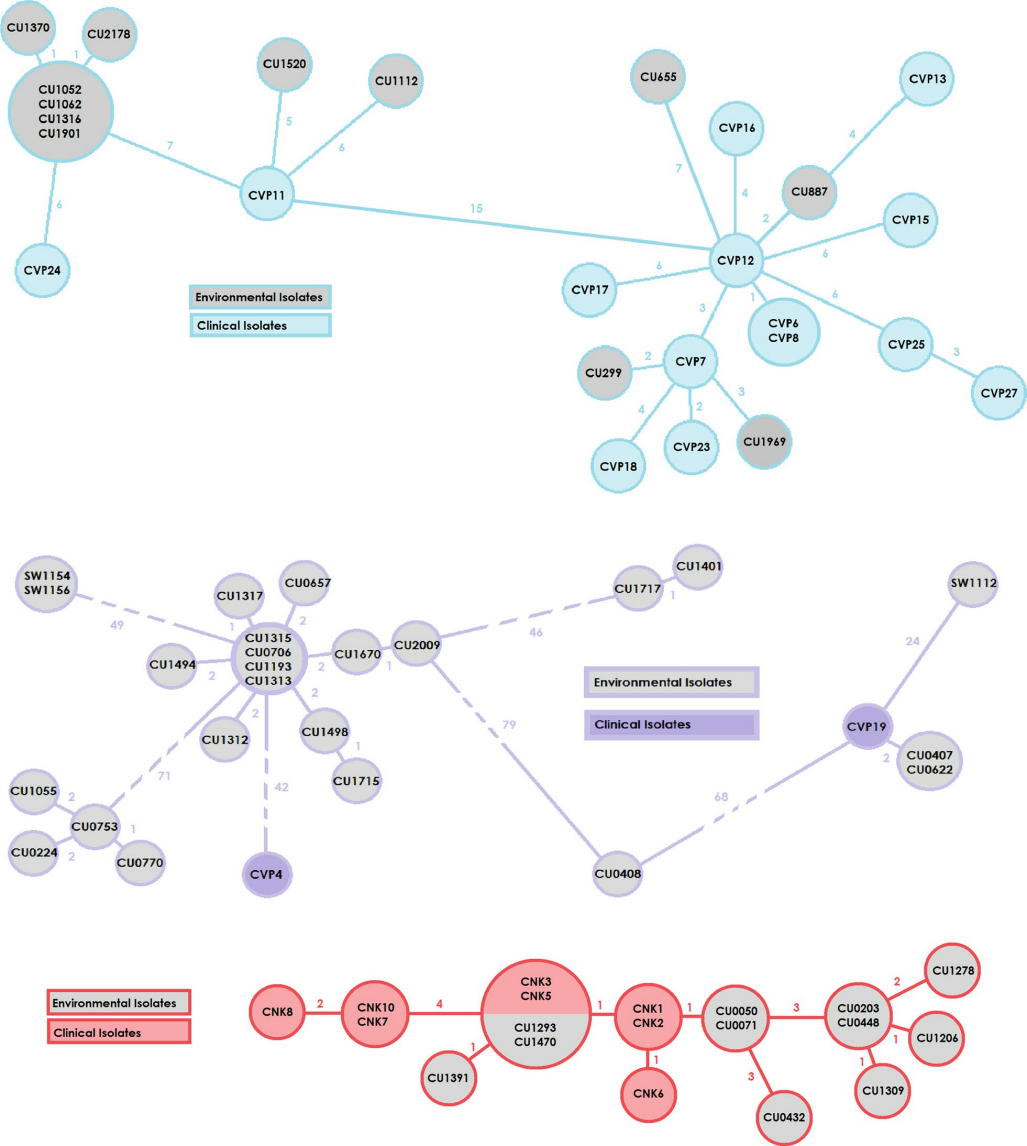
VIM-encoding *P. aeruginosa* ST823 cluster in light blue, VIM-encoding *P. aeruginosa* ST235 cluster in purple, and NDM- and OXA48-encoding *K. pneumoniae* ST147 cluster in red, as minimum spanning tree, respectively from top to bottom. The lines and numbers show the number of allele differences between isolates

Fourteen sequenced *P. aeruginosa* ST823 isolates, fell into two different groups as clustering showed (cluster distance threshold was set to 12) when clustered with environmental isolates from 2016 to 2018 from a study on hospital drains and wastewater [17] (see Fig. 1). Three out of the four environmental isolates in cluster B, which was the cluster with the predominant number of clinical isolates, had been isolated from the drains of patient rooms. Of the two sequenced *P. aeruginosa* ST235 isolates, CVP4 did not cluster with environmental isolates but CVP19 was closely related to two environmental isolates (see Fig. 1). All eight sequenced *K. pneumoniae* ST147 isolates were closely related to each other and to the environmental isolates, forming one large cluster. *P. aeruginosa* ST111 and ST233 isolates and *K. pneumoniae* ST78 isolates were not clustered due to the absence of environmental isolates with matching sequence types.

### Phylogenetic analysis

**Fig. 2:**
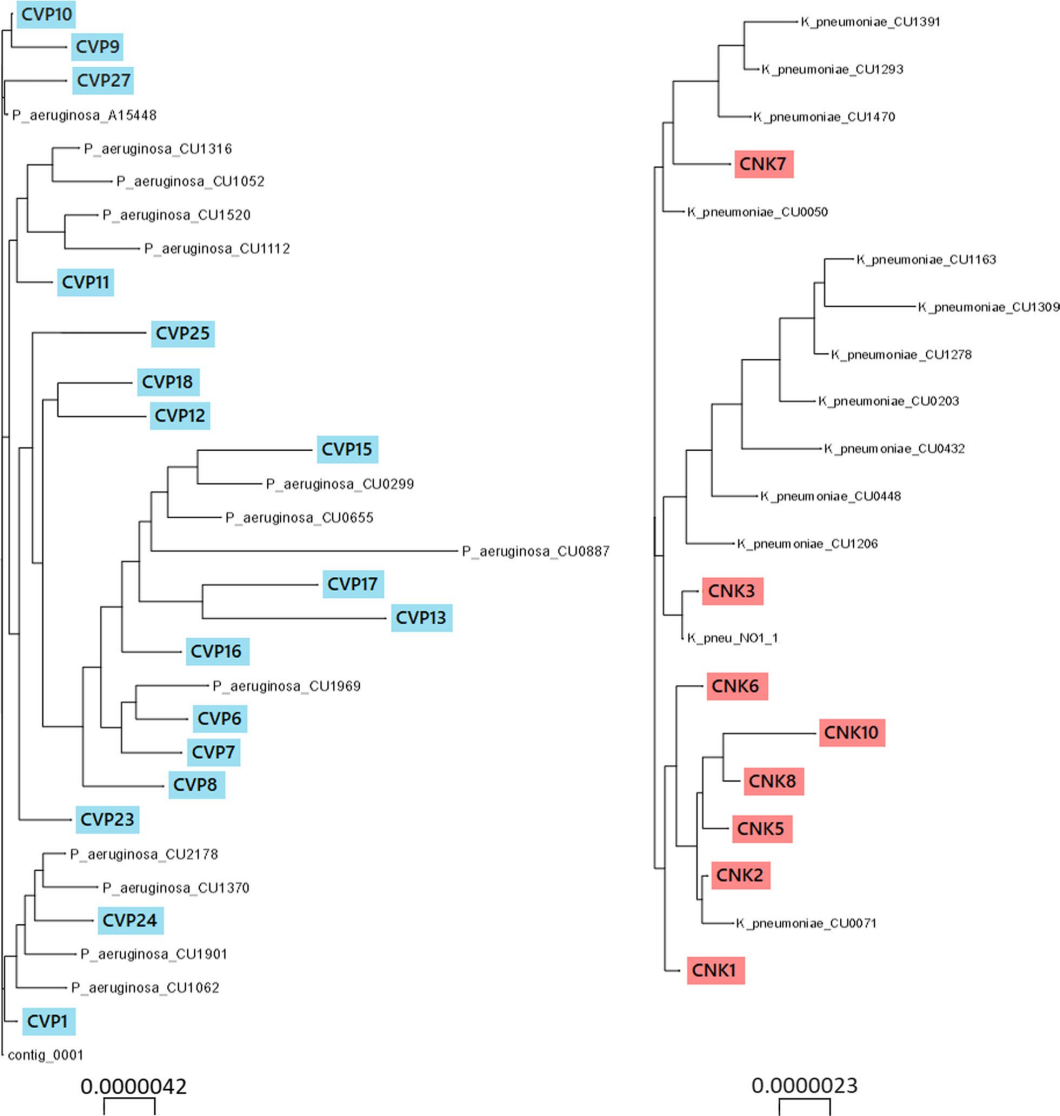
SNP-based maximum-Likelyhood trees of *K. pneumoniae* ST147 and *P. aeruginosa* ST823 isolates (100 bootstraps). Clinical isolates are marked in light blue and red for *P. aeruginosa* and *K. pneumoniae*, respectively

As Fig. 2 shows, the isolates within the *K. pneumoniae* ST147 Cluster exhibited a high degree of relatedness to each other, as did the isolates within the *P. aeruginosa* ST823 cluster. Seven out of eight clinical *K. pneumoniae* ST147 isolates belonged to two very small but extremely tight clusters (CNK7 with environmental isolates CU1391, CU1293, CU1470, and CU0050 and CNK1, CNK2, CNK5, CNK6, CNK8 and CNK10 with the environmental isolate CU0071). The *P. aeruginosa* isolates, on the other hand, even more markedly formed one large cluster with the environmental isolates.

### Resistance genes

**Fig. 3:**
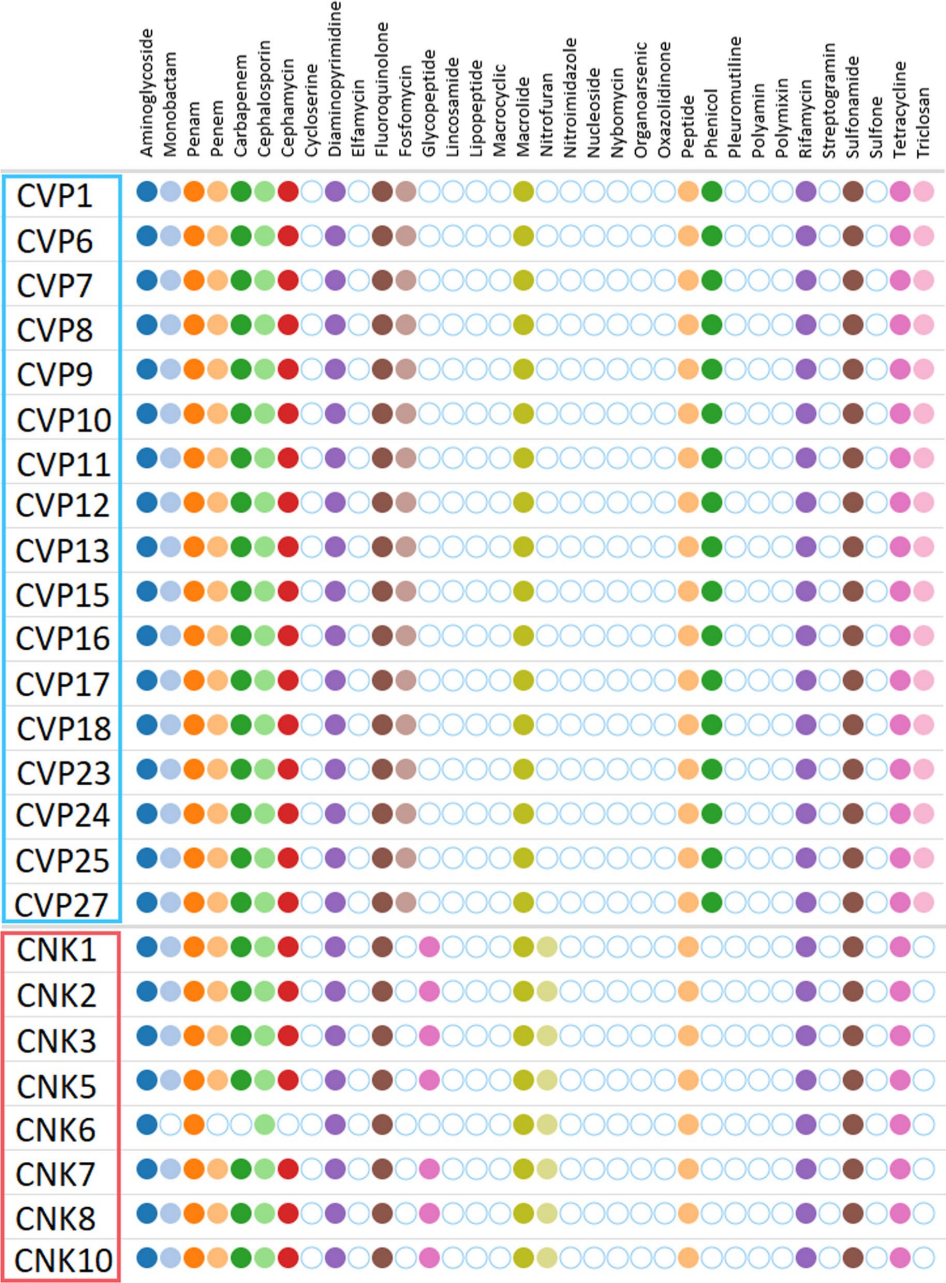
Antibiotic resistances profile as predicted by the ASA^3^P pipeline based on CARD annotations for *P. aeruginosa* ST823 and *K. pneumoniae* ST147 isolates. Colored circles refer to the isolate likely being resistant to the respective substance class, while empty circles refer to the isolate likely being susceptible to it

Plasmid incompatibility groups detected in *K. pneumoniae* isolates by PlasmidFinder-2.0 are listed in Table 3 in the appendix. The table shows that several plasmids were shared with environmental ST147 isolates. *K. pneumoniae* ST78 isolates and ST147 isolates did not share any plasmids. All ST147 isolates carried the same plasmids, except for the three latest ST147 isolates, recovered in 2020 and 2021, which carried an additional IncHI2-type plasmid. Tables 4 and 5 in the appendix show the Antibiotic resistance genes detected on VIM-encoding *P. aeruginosa* genomes and on NDM- and OXA48-encoding *K. pneumoniae* genomes by AMRFinderPlus, respectively. There were only two internal stop codons found with INTERNAL_STOP (CVP2 *fosA* and CVP5 *fosA*) in resistance genes. The VIM-encoding gene that was detected in the course of routine diagnostics in CVP19 was not detected on its sequenced genome, but a repeated targeted PCR with the Allplex Entero-DR Assay (Seegene, Seoul, South Korea) from the isolate and its extracted genomic DNA confirmed the presence of the resistance gene. Predicted antibiotic resistances by the ASA^3^P pipeline for *P. aeruginosa* ST823 and *K. pneumoniae* ST147 isolates are displayed in Fig. 3 in the appendix.

### Phenotypical susceptibility

According to EUCAST 2022 v12 breakpoints (version 01.01, 2022) all tested isolates were resistant to fluoroquinolones and imipenem (see Table 2). Despite the metallo-beta-lactamases (MBL), one *P. aeruginosa* isolate (CVP19) was susceptible at high dosing regimen to meropenem, nine to piperacillin, eleven to piperacillin/tazobactam, and 13 to ceftazidime, as well as 15 at standard dosing regimen to ceftazidime/avibactam. While all *P. aeruginosa* were still susceptible to colistin and 15/22 to ceftazidime/avibactam, 5/10 K*. pneumonia* isolates were resistant to both. Both *K. pneumoniae* ST78 isolates and the three latest ST147 isolates were resistant to colistin. Analysis of the contigs of the IncHI2-type plasmid showed that it did not carry colistin resistance genes; however, the full sequence of the plasmid was not available for analysis. Among these, two ST147 and one ST78 isolate were also resistant to tigecycline. Two ST147 isolates and one ST78 were resistant to chloramphenicol.

**Table 2:**
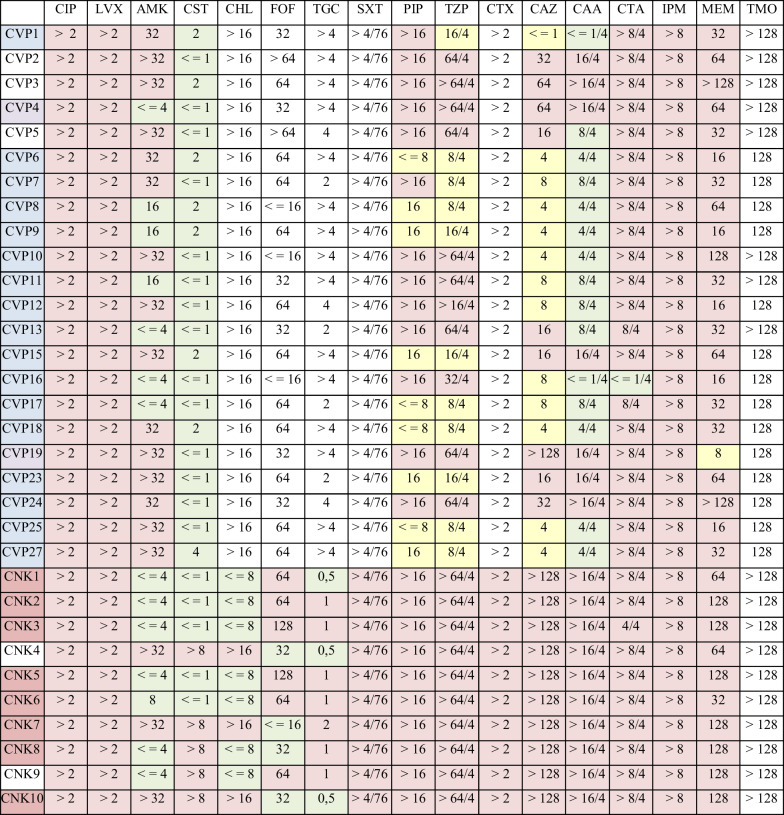
Phenotypical susceptibility of *P. aeruginosa* (above) and *K. pneumoniae* (below) isolates determined by broth microdilution

Isolate cells are colored according to sequence type for better visualization, MIC cells are colored according to isolate susceptibility as per EUCAST 2022 v12 breakpoints (version 01.01, 2022) (green: susceptible, yellow: susceptible at increased dosage, red: resistant)

*CIP*, ciprofloxacin; *LVX*, levofloxacin; *AMK*, amikacin; *CST*, colistin; *CHL*, chloramphenicol; *FOF*, fosfomycin; *TGC*, tigecycline; *SXT*, trimetoprim-sulfamethoxazole; *PIP*, piperacillin; *TZP*, piperacillin/tazobactam; *CTX*, cefotaxime; *CAZ*, ceftazidime; *CAA*, ceftazidime/avibactam; *CTA*, ceftolozan/tazobactam; *IPM*, imipenem; *MEM*, meropenem; *TMO*, temocillin

## Discussion

*K. pneumoniae* clones with high AMR risk represent a tremendous public health burden and have played a central role in the global spread of AMR [33]. *K. pneumoniae* ST147 has emerged as one of the most important AMR clones and clearly exhibits most of the essential characteristics that define a global high-risk AMR clone. Several studies have described efficient transmission between patients in the hospital setting [18] and even through drainage water from one room to another [24, 34]. *P. aeruginosa* is increasingly recognized for the ability of certain hospital populations to cause nosocomial infection outbreaks with significant morbidity and mortality. Both, *K. pneumoniae* and *P. aeruginosa,* form biofilms in toilet bowls, particularly behind the flushing rim of the toilet, and establish themselves in hospital water systems, which allow the pathogens to persist and potentially spread out of the toilet each time it is flushed [35, 36]. The two clustering methods exhibited differences in the exact relationships of the isolates, but both demonstrated that even over years, the patient and environmental isolates clustered very closely together, which supports the assumption that these were the same clones [37, 38].

Our study highlights that, consistently antibiotic-exposed patients might, in interaction with their constantly antibiotic-exposed sanitary facilities form a niche that could be supportive for the emergence of certain nosocomial pathogen populations in the hospital, due to antibiotic-induced selection pressure [11, 39]. These highly resistant clones subsequently survive particularly well in the sanitary facilities of those patients who are at highest risk of colonization or infection, i.e., patients who are frequently treated with broad-spectrum antibiotics due to their immunodeficiency, and who have hardly any remaining healthy normal flora able to outcompete the highly resistant clones [40]. Once colonized, the patients in turn excrete the clones and thus distribute them to other premises and facilities.

In our study, we sequenced two selected sets of carbapenemase-encoding isolates which account only for a fraction of carbapenemase-encoding pathogens encountered in routine practice [41]. While the susceptibility profiles appear atypical for *P. aeruginosa* isolates that encode VIM-type carbapenemases, it should be mentioned that ST823 wastewater isolates exhibited the same kind of antibiograms [17]. Overall, as expected, the therapeutic options were very limited for all isolates [42] with a few newer antibiotics and combinations untested [43]. The effect of the phoQ and pmrB mutations in the strains is still unclear [44–47].Prevention, evidently, remains the much more potent means of combating this problem. Since biofilm is one of the most effective ways for bacteria to colonize the aquatic niche of sanitary facilities, its formation should be prevented. Therefore, several preventive hygienic measures were taken according to national recommendations [48]. As constructional measures, toilets in all newly build wards are designed rimless and in high-risk areas toilets were remodeled to fit the rimless standard. As additional routine hygienic measures, surfaces of toilets, sinks and shower basins are daily disinfected chemically, moreover, sink and shower drains are incubated with a solution of oxidizing disinfectants on a weekly basis, in order to minimize biofilm formation and to reduce high microbial burden. In high risk areas (e.g. bone marrow transplantation) disinfection devices on sink drains applying heat and electromechanical vibration [49] had been installed, however, regarding toilets and shower basins there exist no corresponding technical solutions [48]. With the implemented hygiene and prevention measures presumably colonization pressure is decreased due to reduced microbial load of showers and sinks. The present study did not involve an individualized, patient-specific evaluation of transmission routes or a clinical assessment. It is highly probable that all the following transmission routes existed: from patient to environment, from environment to patient, directly from patient to patient, and through cases that were imported and became detectable under antibiotic selection pressure. Without more detailed analyses, it is not possible to accurately determine which transmission route dominated, only that all of these possibilities exist.

As for the differences between genotype-predicted/expected and phenotypic resistance, it must be pointed out that the accuracy of such algorithms decreases with the number of resistance and virulence genes present, which the isolates analyzed here have an overabundance of. Moreover, it is particularly difficult to infer resistance from the genotype in *P. aeruginosa*, as resistance is usually porin-mediated rather than resistance gene-mediated [50]. For example, *fosA* was detected on all *P. aeruginosa* genomes, whereas a few were phenotypically susceptible, yet, these were not the two isolates in which the gene was determined to be non-functional. Thus, despite the many advantages of molecular antibiograms [51], conventional resistance testing appears to be indispensable, especially for such highly resistant pathogens that are increasingly being screened for molecularly.

## Conclusions

Hospital drains continue to play a role in the spread of multidrug-resistant pathogens, as they might form favorable niches for the emergence of multidrug-resistant bacterial populations influenced significantly due to the constant patient-driven antibiotic selection pressure. Extensive technical (e.g. rimless toilets) and hygienic measures (i.e. chemical or technical disinfection of drains), constant monitoring and strict hygiene precautions help to prevent infections, however, further technical solutions are needed to prevent biofilm formation and selection pressure at the sanitary inventory level, as antibiotics are necessary for therapy, but their metabolites in the drains cause undesirable effects.

## Figures and Tables

**Table 3:**
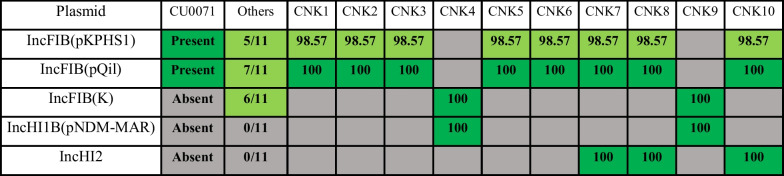
Plasmids detected in NDM- and OXA48-encoding *K. pneumoniae* isolates by PlasmidFinder-2.0

Numbers in cells refer to sequence identity and are colored for easier visualization (for CNK isolates: dark green for 100% sequence identity, light green for 98.57% sequence identitiy; for CU0071 dark green for presence, grey for absence of the plasmid; for others: light green for the presence of the plasmid in some of the isolates, grey for the absence in all). The closely related environmental isolates (others) and CU0071 in particular [17] are included in the table to visualize shared plasmids. The “others” column refers to how many of the “other” environmental isolates carried the respective plasmid

**Table 4:**
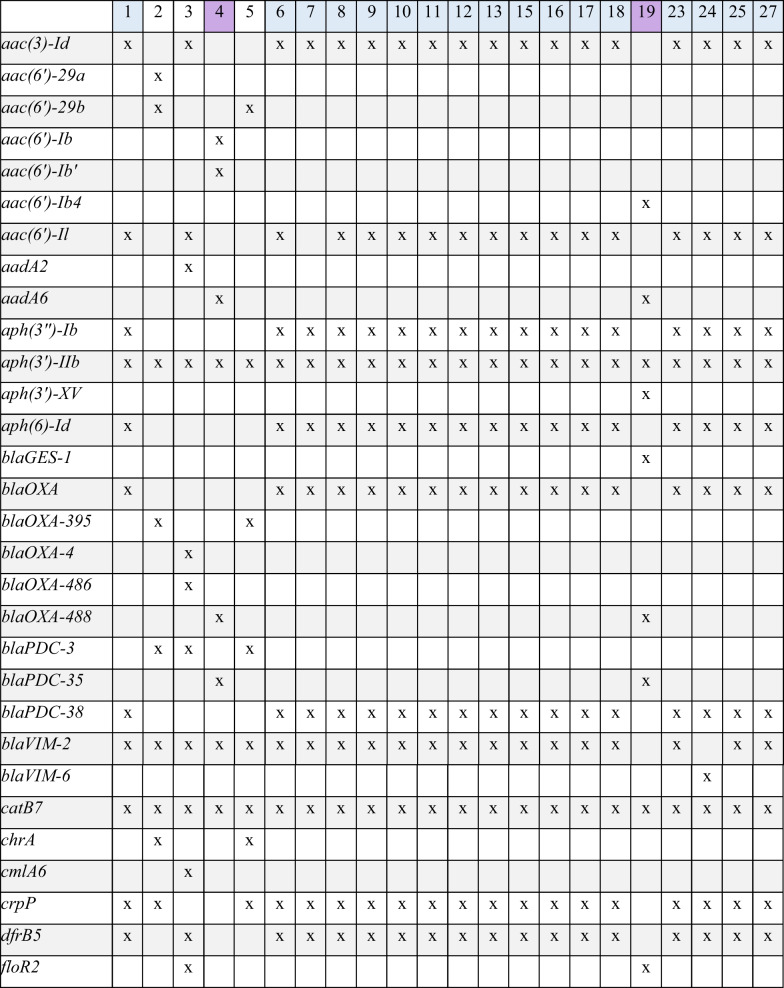

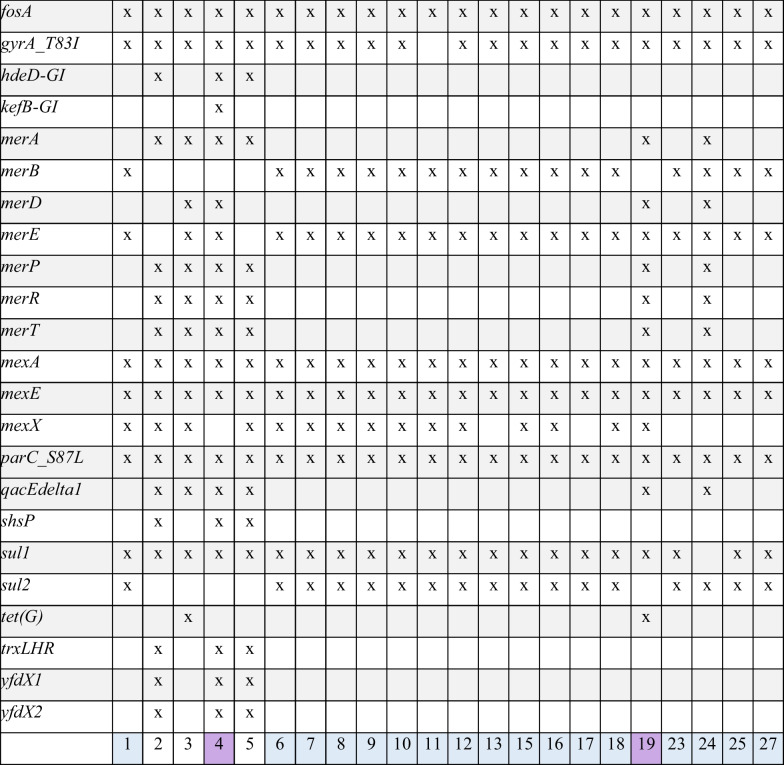
Antibiotic resistance genes detected on VIM-encoding *P. aeruginosa* genomes by the AMRFinder

Isolates are colored according to isolate sequence type as in previous tables and figures

**Table 5:**
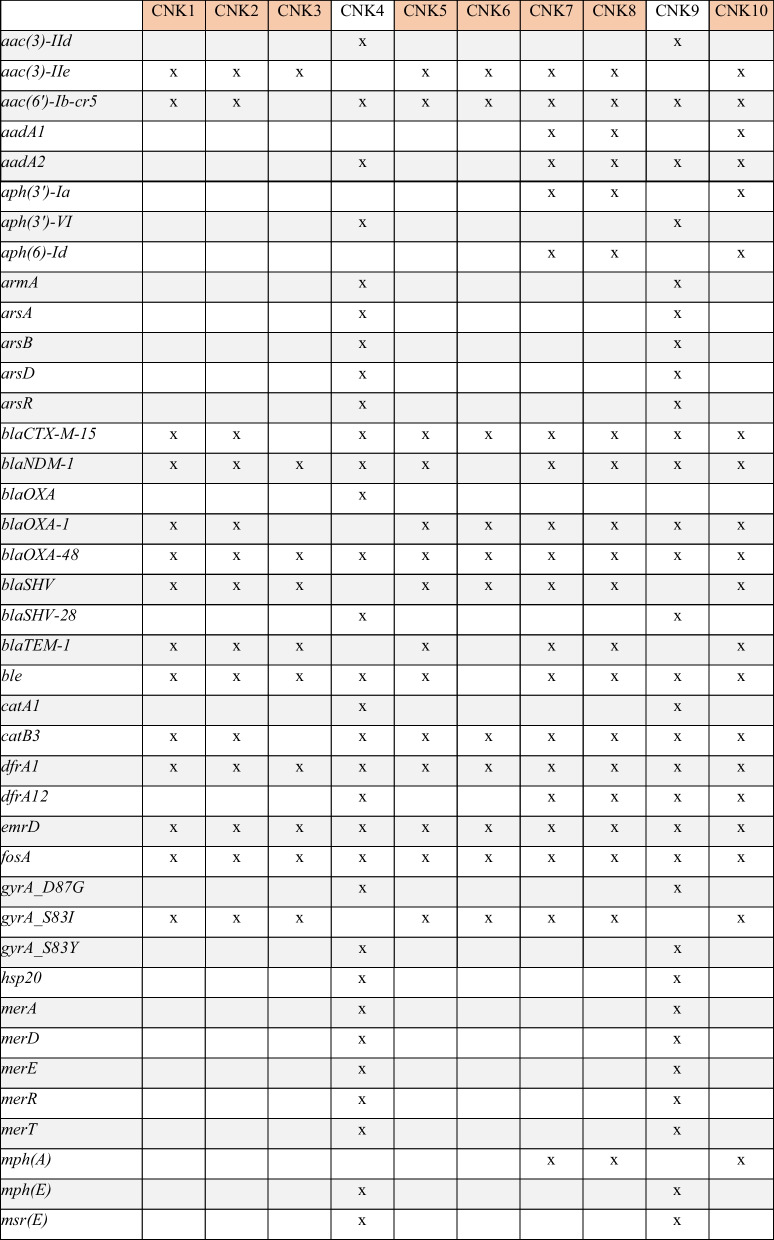

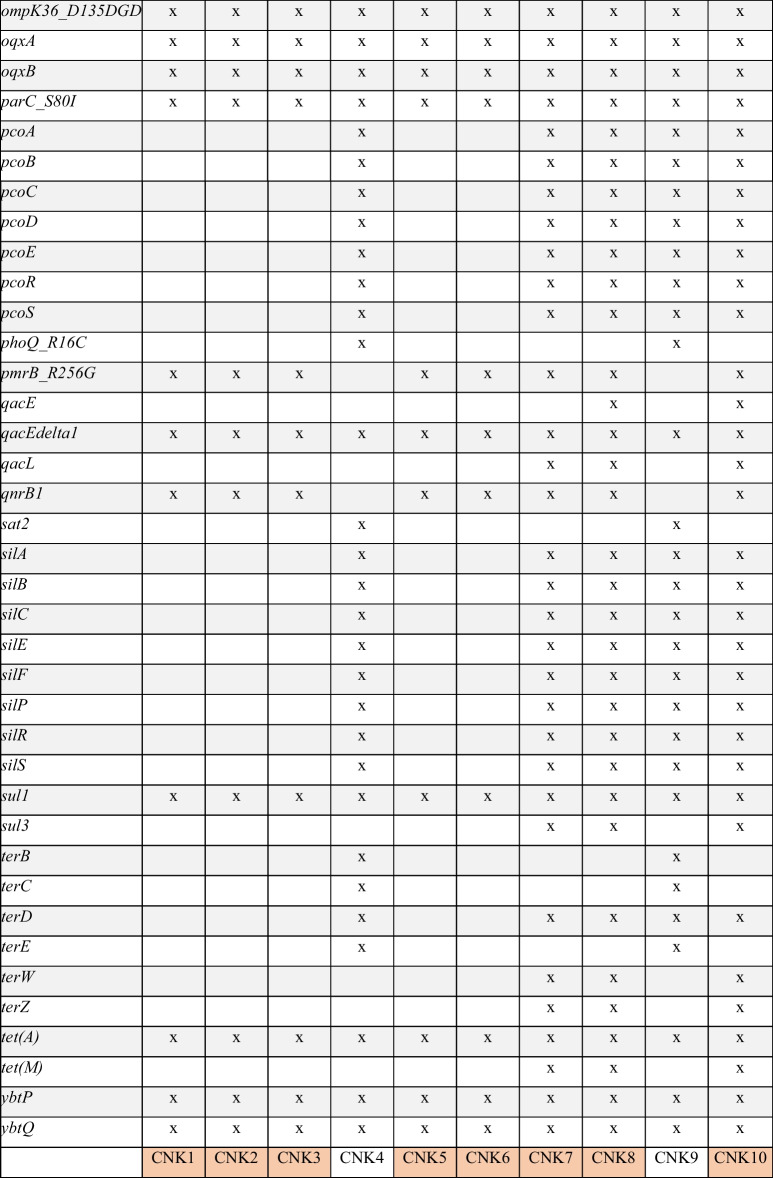
Antibiotic resistance genes detected on NDM- and OXA48-encoding *K. pneumoniae* genomes by the AMRFinder

Isolates are colored according to isolate sequence type as in previous tables and figures

## Data Availability

All data relevant to the study are included in the article or uploaded as Appendix. Raw reads have been uploaded to the Sequence Read Archive (SRA); accession PRJNA845217.

## Appendix

(See Fig. 3 and Tables 3, 4, 5).
